# Fruquintinib and sintilimab plus SOX as perioperative therapy for locally resectable advanced gastric/gastroesophageal junction adenocarcinoma: study protocol for a prospective, single-arm, phase II clinical trial

**DOI:** 10.3389/fimmu.2025.1638316

**Published:** 2025-07-29

**Authors:** Xiangyu Meng, Dong Yang, Yuanlin Liu, Chao Wang, Junqiao Yao, Tao Zhang

**Affiliations:** Department of Gastric Surgery, Cancer Hospital of China Medical University/Liaoning Cancer Hospital, Shenyang, Liaoning, China

**Keywords:** G/GEJ adenocarcinoma, perioperative treatment, fruquintinib, sintilimab, effectiveness

## Abstract

**Background:**

Locally advanced gastric/gastroesophageal junction (G/GEJ) adenocarcinoma faces high recurrence risks despite radical surgery. Perioperative chemotherapy (e.g., FLOT regimen) improves survival but has limited pathological complete response (pCR) rates and significant toxicity. Immunotherapy and anti-angiogenic agents show promise in advanced G/GEJ cancer. This trial evaluates fruquintinib (a VEGFR-1/2/3 inhibitor), sintilimab (PD-1 inhibitor), and SOX (oxaliplatin+S-1) as perioperative therapy for resectable locally advanced G/GEJ adenocarcinoma.

**Methods:**

This prospective, single-arm, phase II trial (N = 25) enrolls treatment-naïve adults (18–75 years) with histologically confirmed, resectable cT3-4aN+M0 G/GEJ adenocarcinoma (AJCC 8th edition). Patients receive 3 cycles of neoadjuvant therapy:Fruquintinib:4 mg orally, days 1–14 (21-day cycle). S-1: 80–120 mg orally twice daily (based on BSA), days 1–14. Oxaliplatin: 130 mg/m² IV, day 1. Sintilimab: 200 mg IV, day 1.Radical gastrectomy with D2 lymphadenectomy follows 4–6 weeks post-neoadjuvant therapy. Adjuvant therapy (3 cycles of sintilimab for pCR patients; 3 cycles of preoperative regimen for non-pCR) starts 4–6 weeks post-surgery. Endpoints: Primary: pCR rate (ypT0/Tis ypN0 per CAP criteria). Secondary: R0 resection rate, major pathological response (MPR, ≤10% residual tumor), 2-year event-free survival (EFS), 2-year overall survival (OS), safety (NCI CTCAE v5.0). Exploratory: Biomarker analysis of tumor microenvironment. Statistical Analysis: Sample size (25 patients) was calculated using Fisher’s exact test (one-sided α = 0.05, power = 80%), assuming pCR improvement from 5% (historical control) to 20%. Efficacy analyses use intention-to-treat (ITT) population; safety analyses include patients receiving ≥1 neoadjuvant dose.

**Discussion:**

This is the first trial combining fruquintinib, sintilimab, and SOX in perioperative G/GEJ cancer. If successful, it may expand treatment options for locally advanced disease. Limitations include single-arm design and small sample size.

**Trial Registration:**

Chinese Clinical Trial Registry (ChiCTR2400084194)

## Introduction

Gastric and gastroesophageal junction (G/GEJ) cancer continue to represent a significant global health burden, maintaining their status as the fourth most prevalent oncologic diagnosis and the fourth leading contributor to cancer-associated mortality. Epidemiological data from 2022 reveal a staggering disease incidence exceeding 1 million newly diagnosis (1). While radical gastrectomy remains the cornerstone of curative intervention for G/GEJ cancer, clinical outcomes are paradoxically challenged by substantial recurrence and metastatic risks, particularly in patients with locally advanced carcinoma (clinical stage ≥T3 or node-positive disease) (2). Neoadjuvant therapy (NAC) has emerged as a pivotal component of multimodal cancer management, demonstrating proven efficacy in achieving pathological downstaging, mitigating micrometastatic dissemination, enhancing R0 resection rates through tumor volume reduction, and significantly improving long-term survival outcomes (3–5). Several guidelines also recommend NAC in locally advanced gastric cancer (LAGC) (6–10). The CLASSIC trial and the ACTS-GC trial have demonstrated that adjuvant chemotherapy provided a survival benefit compared with observation alone following gastrectomy with D2 lymph node dissection (10–12). The MAGIC trial and FLOT4 trial established and optimized perioperative chemotherapy models by pioneering the multimodal perioperative strategy with epirubicin-cisplatin-fluorouracil (ECF) and FLOT triplet therapy in Western populations with resectable gastric/gastroesophageal adenocarcinoma (12, 13). Therefore, the combination of neoadjuvant therapy, radical surgery, and adjuvant therapy has become the standard treatment modality for LAGC.

Immunotherapy has reshaped treatment modality for advanced or metastatic G/GEJ cancer patients. Thecheckmate-649 study shows that the combination of Nivolumab and chemotherapy increase the duration of PFS and OS in patients with CPS≥5 and CPS≥1 compared with chemotherapy alone, and a statistical difference is seen in the entire population (13.8 months versus 11.6 months, HR=0.80) (14). The results of ATTRACTION-4 shows that the median progression-free survival (PFS) time (10.5 months: 8.3 months, HR=0.68) and objective response rate (ORR) (57.5% *vs* 47.8%, P=0.0088) are significantly better than those of chemotherapy alone (15). The results of the Checkmate649 and ATTRACTION-4 studies confirm the role of immunochemotherapy in the first-line treatment of advanced or metastatic G/GEJ cancer patients. The results of the ORIENT-16 trial demonstrated that sintilimab, a programmed death 1 (PD-1) inhibitor, in combination with the XELOX regimen, is both safe and effective, which enhances the OS of metastatic gastric cancer patients with CPS≥5 (16). Although many guidelines do not endorse the incorporation of PD1/PD-L1 inhibitors into NAC, several studies have demonstrated that the integration of immunotherapy into NAC may enhance the efficacy of treatment for locally advanced gastric cancer. The KEYNOTE-585, MATTERNHORN and DANTE trials demonstrate that the combination of PD-L1 inhibitors with chemotherapy significantly enhance the pathological complete response (pCR) rate in patients with LAGC compared to chemotherapy alone (17–19). However, it remains to be seen whether the improved pCR observed in these trials will translate into a survival benefit.

Vascular endothelial growth factor (VEGF), a homodimeric signaling protein, is constitutively expressed by multiple cell lineages under both homeostatic and pathophysiological conditions. As cognate receptors for VEGF ligands, VEGFRs demonstrate characteristic receptor tyrosine kinase (RTK) activation through ligand-induced dimerization, with the tripartite family (VEGFR-1/2/3) differentially regulating angiogenesis (VEGFR-2), vascular maturation (VEGFR-1), and lymphangiogenesis (VEGFR-3) (20, 21). VEGFR-tyrosine kinase inhibitors including apatinib have shown excellent clinical efficacy in the treatment of advanced or metastasis gastric cancer (22). Some studies have also shown that apatinib combined with SOX/XELOX regimen can significantly improve the major pathological response (MPR) rate of LAGC patients compared with chemotherapy alone (23, 24). The Dragon IV trial demonstrates that the combination of apatinib, PD-1 inhibitors, and chemotherapy significantly enhanced the pCR rate in the perioperative treatment of locally advanced gastric cancer compared to chemotherapy alone (18.3% *vs* 5%) which suggests the feasibility of using small molecule TKI combined with multimodal therapy in LAGC (25). However, the Dragon IV trial also showed that apatinib combined with PD1 and SOX chemotherapy also increased the side effects compared with SOX chemotherapy (≥grade 3 treatment-related AEs data: 34% vs. 17%) (25). Fruquintinib, a highly selective small molecule inhibitor of VEGFR-1, VEGFR-2 and VEGFR-3,is an orally available VEGFR inhibitor and widely used in the third-line treatment of metastatic colorectal cancer (CRC) (26). There is limited evidence regarding the use of fuquinitinib in G/GEJ) cancer. Only a few small sample data have shown the prospect of fuquinitinib in the treatment of G/GEJ cancer (27). The FRUTIGA study, a randomized, double-blind, phase III trial, is evaluating the efficacy and safety of fruquintinib combined with paclitaxel for advanced GC/GEJ patients who did not respond to first line standard chemotherapy (NCT03223376) (28, 29). The trail indicates that Fruquintinib in combination with paclitaxel demonstrates a significant improvement in progression-free survival (PFS) compared to paclitaxel monotherapy, and may serve as a novel second-line treatment option for Chinese patients with advanced. In the safety assessment, in colon cancer, FRESCO trail confirmed lower rates of grade ≥ 3 hypertension (21.2%) and proteinuria (2.6%) with furoquininib than in the same class (apatinib: hypertension 39.9%) without cumulative toxicity (26). A single-arm, open-label phase II study of fuquinitinib in combination with PD1 (toripalimab) and chemotherapy (XELOX/SOX) in locally advanced unresectable or metastatic G/GEJ showed that 22.2% (6/27) experienced grade 3 adverse events (NCT06158919). Another single-arm, phase 2 study of fuquinitinib plus sintilimab and chemotherapy for conversion therapy in G/GEJ) cancer showed that the incidence of grade 3 or above treatment-related AEs was only 16.7% (7/42). No severe surgery-related complication was observed (NCT05177068). These trials involving furoquininib plus PD1 and single-agent or two-agent chemotherapy showed a lower incidence of grade 3 or higher adverse events than the 34% observed in the Dragon IV study. Therefore, the safety of furoquininib combined with PD1 and SOX regimen used in this study in the perioperative treatment is worthy of expectation.

In summary, in order to enrich the perioperative treatment regimen of LAGC and improve the prognosis of patients with LAGC, the aim of this study is to evaluate the safety and efficacy of fuquinitinib combined with sintilimab combined with SOX regimen in the perioperative treatment of locally advanced GC/GEJ cancer.

## Methods and analysis

**Figure 1:**
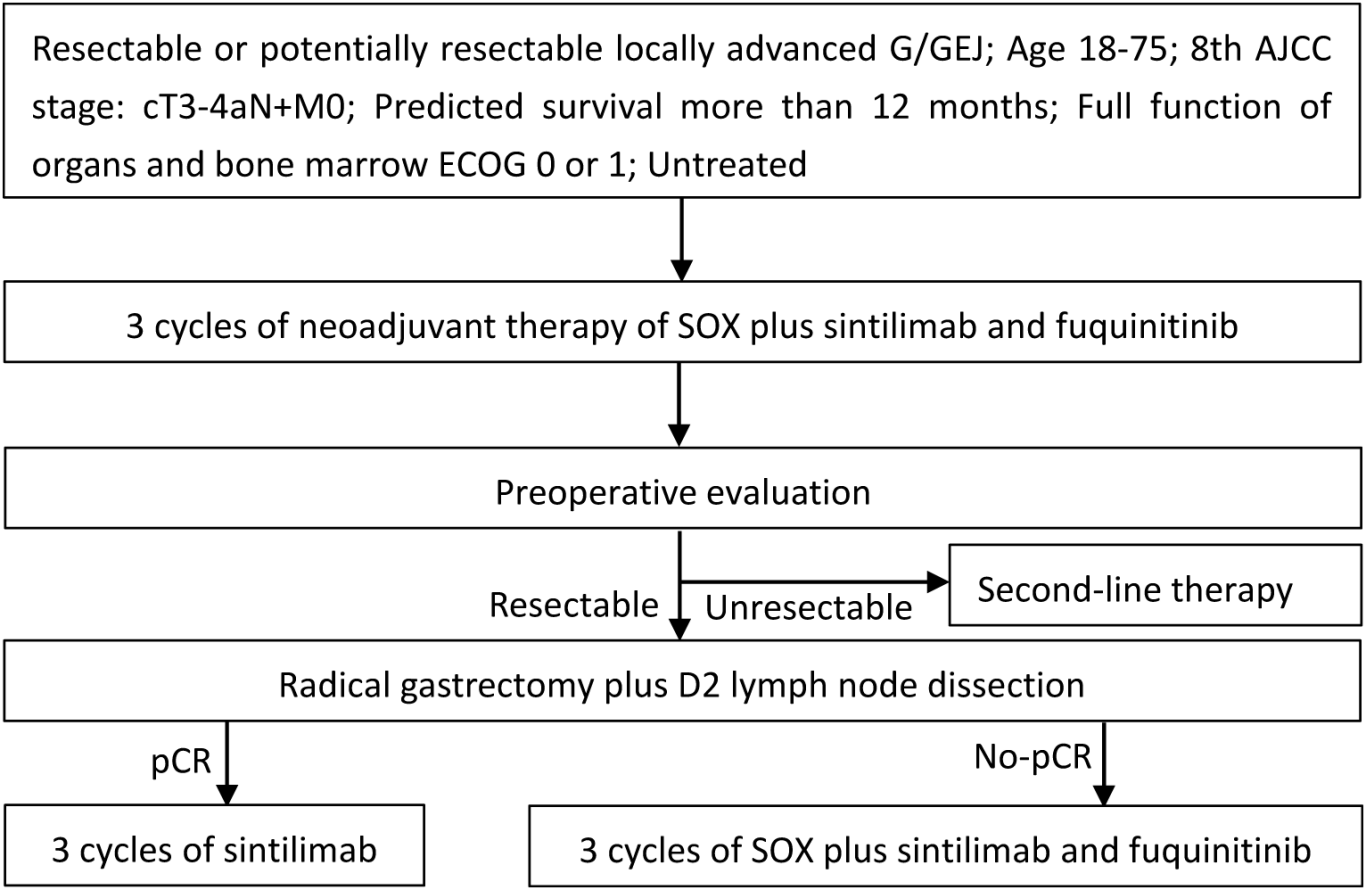
Study flow chart.

This prospective, single-arm, phase II trial was designed to assess the efficacy and safety of SOX combined with fuquinitinib and sintilimab in the perioperative treatment of resectable locally advanced G/GEJ cancer. The study protocol and the informed consent forms have been reviewed and approved by the Research Ethics Committee of the Liaoning Tumor Hospital & Institute (approval number 2024350). This study will be conducted in strict compliance with the ethical principles of the Declaration of Helsinki and the International Council for Harmonization of Technical Requirements for Pharmaceuticals for Human Use (ICH) Good Clinical Practice (GCP) guidelines. All subjects enrolled in the study must provide written informed consent, and all data will be handled confidentially. The study has been prospectively registered at www.chictr.org.cn (ChiCTR2400084194). A schematic diagram of the study design is shown in **Figure 1**.

### Endpoints

The primary endpoint is pathological complete response (pCR) rate, defined as the absence of viable tumor cells (ypT0/Tis ypN0) in both primary and lymph node specimens confirmed by histopathological assessment per College of American Pathologists (CAP) criteria following neoadjuvant therapy. Secondary endpoints encompass R0 resection rate (microscopically margin-negative resection of residual tumor), major pathological response (MPR) rate (≤10% residual viable tumor cells in the primary lesion and regional lymph nodes), 2-year event-free survival (EFS), 2-year overall survival (OS), and safety parameters, with adverse events (AEs) graded using NCI Common Terminology Criteria for Adverse Events (CTCAE) v5.0 and serious adverse events (SAEs) defined as fatal/life-threatening outcomes, hospitalization/prolonged hospitalization, persistent disability, congenital anomalies, or clinically significant complications requiring intervention. Exploratory endpoints (EBV,CPS score, and Microsatellite status) was used to evaluate the multimodality treatment benefit population with help of stratified analysis based on biomarkers. Meanwhile, multiplex immunofluorescence (mIF), and RNA sequencing (RNA-seq) from tumor tissue before treatment and after surgery will be analysis to explore the tumor microenvironment profiling.

### Study population and eligibility criteria

The key inclusion criteria include the following:

1. Age ≥18 and ≤75 years at screening.
2. Histologically confirmed G/EGJ cancer.
3. Clinically resectable (T3-4aN+M0) per AJCC 8th edition staging confirmed by contrast-enhanced CT.
4. Treatment-naïve status (no prior anticancer therapy including surgery, radiotherapy, chemotherapy, targeted therapy, immunotherapy, etc.).
5. Planned definitive surgical resection following neoadjuvant therapy completion.
6. Capacity for oral medication intake without dysphagia.
7. ECOG PS 0-1.
8. Anticipated life expectancy ≥12 months.
9. Adequate organ function meeting all criteria below:

• Hematological (without transfusion/G-CSF support within 14 days):

ANC ≥1.5×10^9^/L; Platelets ≥80×10^9^/L; Hemoglobin ≥80g/L.

• Hepatic/Renal:

Total bilirubin <1.5×ULN; ALT/AST ≤2.5×ULN; Serum creatinine ≤ 1.5 × ULN or CrCl >50 mL/min (Cockcroft-Gault formula: Male: CrCl = ((140 - age) × weight)/(72 × SCr); Female: CrCl = ((140 - age) × weight)/(72 × SCr) × 0.85 (Weight in kg; SCr in mg/dL)).

The key exclusion criteria include were as follows:

1. Known HER2 overexpression.
2. Allergic to fruquintinib, oxaliplatin, or tegafur/gimeracil/oteracil (S-1).
3. Prior use of anti-angiogenic small molecule TKIs (e.g., regorafenib, apatinib, lenvatinib, anlotinib) other than fruquintinib within the screening period.
4. Prior treatment with fruquintinib, oxaliplatin, or tegafur/gimeracil/oteracil (S-1).
5. Esophagogastric junction (EGJ) carcinoma involving the proximal stomach with tumor epicenter ≤2 cm from the EGJ.
6. Known peritoneal metastasis, positive peritoneal cytology (CY1P0), or T4b disease (per AJCC 8th edition staging).
7. Unresectable disease due to tumor characteristics, surgical contraindications, or patient refusal of surgery.
8. History of concurrent or prior malignancies, except cured basal cell carcinoma, cervical carcinoma *in situ*, or breast carcinoma *in situ*.
9. Poorly controlled hypertension despite antihypertensive therapy (systolic BP ≥140 mmHg or diastolic BP ≥90 mmHg).
10. Cardiac abnormalities meeting any of the following:
  • NYHA Class II or higher heart failure, or LVEF <50% by echocardiography.
  • Unstable angina.
  • Myocardial infarction within 1 year.
  • QTc >450 ms (male) or >470 ms (female) on resting ECG.
  • Clinically significant ECG abnormalities (e.g., conduction defects, complete left bundle branch block, third-degree AV block, second-degree AV block, or PR interval >250 ms).
  • Risk factors for QTc prolongation (e.g., heart failure, hypokalemia, congenital long QT syndrome, family history of sudden death <40 years, concomitant QT-prolonging medications).
11. History of gastrointestinal perforation, intra-abdominal abscess, intestinal obstruction within 3 months, or clinical/imaging findings suggestive of obstruction.
12. Coagulopathy (INR>2.0 or PT>16s), bleeding diathesis, or current thrombolytic/anticoagulant therapy (prophylactic low-dose aspirin or LMWH permitted).
13. Clinically significant bleeding within 3 months (e.g., gastrointestinal hemorrhage, hemorrhagic gastric ulcer) or persistent fecal occult blood positivity requiring gastroscopy (unless ruled out by gastroscopy within 3 months).
14. Arterial/venous thromboembolic events within 6 months (e.g., stroke, transient ischemic attack, DVT, pulmonary embolism).
15. Known hereditary or acquired bleeding/thrombotic disorders (e.g., hemophilia, thrombocytopenia).
16. Active ulcers, unhealed wounds, or fractures.
17. Urine protein ≥++ on dipstick with 24-hour urine protein >1.0 g.
18. Active infection requiring antimicrobial therapy (antibacterial, antiviral, or antifungal agents).
19. Active hepatitis (HBsAg-positive with HBV DNA ≥500 IU/mL; HCV antibody-positive with HCV RNA >ULN).
20. Congenital or acquired immunodeficiency (e.g., HIV infection).
21. Prior or planned organ/allogeneic bone marrow transplantation.
22. Interstitial lung disease (ILD) or history of steroid-requiring ILD; pulmonary fibrosis, organizing pneumonia, active pneumonia, severe pulmonary dysfunction on CT, or active tuberculosis.
23. Active autoimmune disease or history of autoimmune disease with relapse risk (e.g., autoimmune hepatitis, uveitis, thyroiditis; exceptions: vitiligo, psoriasis, alopecia, well-controlled hypothyroidism on hormone replacement, childhood asthma in remission).
24. Systemic immunosuppressants or corticosteroids (>10 mg/day prednisone equivalent) within 7 days.
25. Live attenuated vaccination within 28 days or planned during study.
26. Strong CYP3A4 inducers within 2 weeks or inhibitors within 1 week prior to randomization.
27. Participation in other clinical trials within 4 weeks.

### Intervention

Patients who meet the eligibility criteria will be enrolled and signed the informed consent form. All eligible patients will be registered and enrolled to undergo 3 courses of neoadjuvant treatment: Fruquintinib will be orally administered once daily at a dose of 4mg during days 1–14 of a 21-day cycle; S-1 will be orally administered twice a day, with the dosage determined by the patient’s body surface area (<1.25m^2^, 80 mg; ≥1.25 to <1.5m^2^, 100 mg; ≥1.5m^2^, 120 mg/day) on days 1–14 of a 21-day cycle; Oxaliplatin, at a dose of 130 mg/m^2^, will be intravenously administered on day 1 every 3 weeks; Sintilimab: at a dose of 200mg, will be intravenously administered on day 1 every 3 weeks. Radical gastrectomy plus D2 lymph node dissection will be performed 4–6 weeks after the last administration of the SOX plus Fruquintinib and sintilimab regimen. Adjuvant therapy will start in 4–6 weeks after surgery. For patients achieving pathologic complete response (pCR), 3 cycles of sintilimab (200 mg intravenously on Day 1 or 2 of each 21-day cycle) will be administered. For non-pCR patients, a three-cycle preoperative treatment will be performed.

### Toxicity monitoring and dose adjustments

Toxicity will be evaluated prior to each treatment cycle based on patient history, physical examination, and laboratory assessments, including complete blood count, liver function tests, and renal function tests. AEs will be classified according to the NCI CTCAE v5.0. Dose modifications will be determined by the type and severity of the observed toxicities. For chemotherapy-associated toxicities (e.g., myelosuppression, nausea, vomiting, diarrhea), concurrent dose reductions of S-1 will be applied. In cases of severe hematologic toxicity (e.g., grade ≥3 neutropenia or thrombocytopenia), both S-1 and oxaliplatin will be reduced by one dose level, and treatment will be postponed until recovery if toxicity remains unresolved. The minimum recommended daily dose for S-1 is 60 mg, and the minimum dose for oxaliplatin is 85 mg/m². For fruquintinib-related toxicities (e.g., hand-foot syndrome, hypertension, fatigue, liver dysfunction), the recommended dose will be reduced to 3 mg/day in the event of grade ≥3 toxicities, with a further reduction to 2 mg/day if clinically indicated. Temporary treatment interruptions may be necessary for persistent adverse events, and resumption of therapy will be contingent upon adequate recovery. When sintilimab is administered, the following immune-related adverse events-such as pneumonitis, enteritis, hepatitis, nephritis, endocrine disorders, dermatitis, myocarditis, and neurotoxicity-are assessed as grade 2. In such cases, medication should be discontinued. Sintilimab should be resumed in patients with improvement to grade 0–1 after tapering of corticosteroids or stabilization of hormone replacement therapy. Treatment was permanently discontinued in cases where any of these immune-related adverse effects reached grade 3 or higher, failed to improve to grade 0–1 within 12 weeks following the initiation of steroid therapy, or if prednisone could not be tapered to ≤10 mg per day (or equivalent) within the same time frame. For other toxicities that are difficult to classify, dose modifications will be guided by the investigator’s clinical judgment to ensure patient safety and continuity of treatment. Discontinuation of the study regimen will be considered if such toxicities persist despite two dose reductions, if intolerable adverse events recur, or if patients withdraw consent or demonstrate disease progression. Supportive care measures will include the use of antiemetics for nausea and vomiting, antihypertensive agents for elevated blood pressure, specialized dermatologic care for hand-foot syndrome, and general supportive interventions for fatigue and other associated symptoms.

### Follow-up

All patients will undergo scheduled follow-up assessments every 3 months (± 7 days) for survival monitoring and tumor status evaluation within the first 3 years post-surgery. After this initial period, assessments will transition to every 6 months (± 7 days) until meeting any of the following endpoints: documented death, loss to follow-up, withdrawal of informed consent, refusal to provide further data, or trial termination. Tumor evaluations will primarily rely on standard imaging modalities (e.g., CT/MRI), with supplementary methods such as endoscopic ultrasound (EUS) or FDG-PET/CT implemented if clinically indicated. For patients discontinuing study treatment without evidence of disease progression or death, tumor assessments will continue per protocol until radiographic confirmation of progression. The total study duration is projected to span 3 years from enrollment completion to final analysis.

### Statistical analysis

The primary endpoint of this study is pCR rate. The sample size was determined based on the following assumptions: a one-sided alpha level of 0.05, 80% statistical power (beta=0.2), and an expected improvement in pCR rate from 5% (historical control from the phase III RESOLVE trial (4) to 20% in our cohort. Using Fisher’s exact test for proportion comparison in SPSS V.25.0 software (IBM) and R software V.3.6.2 (http://www.R-project.org), the initial calculation yielded a required sample size of 22 patients. Accounting for a potential 10% dropout rate and clinical enrollment feasibility, the final target enrollment was set at 25 participants. Descriptive statistics will summarize baseline demographics and clinicopathological characteristics. Efficacy endpoints will be calculated with corresponding 95% confidence intervals (CIs) using Blaker’s binomial exact method. EFS and overall survival OS will be evaluated through Kaplan-Meier estimates, accompanied by 95% CIs. Primary analyses will utilize the intention-to-treat (ITT) population. Safety analyses will focus on patients receiving ≥1 dose of neoadjuvant therapy (safety population). Neoadjuvant-and adjuvant-related emergent adverse events (AEs) will be stratified by treatment phase due to regimen differences. Surgical outcomes (morbidity/mortality) will be assessed in the per-protocol population, defined as protocol-compliant patients undergoing surgery. Subgroup analysis of biomarkers: the pCR rates of patients with PD-L1 CPS≥ 5 *vs <*5, and MSI-H *vs* MSS were compared (Fisher’s exact test), and the OR value and 95%CI were calculated. To mitigate missing data, proactive measures were implemented during trial design, including standardized data collection protocols. For participants with missing outcome data due to withdrawal or loss to follow-up, multiple imputation techniques will be employed.

## Discussion

The advent of landmark trials such as MAGIC (30) and FLOT (13) has established perioperative therapy combined with radical surgery as a cornerstone in the comprehensive management of LAGC. Compared to surgery followed by adjuvant chemotherapy alone, the incorporation of preoperative chemotherapy demonstrates significant survival benefits, particularly in improving OS for locally advanced disease. Evidence from East Asian population-based studies including PRODIGY (3) and RESOLVE (4) further supports the gradual adoption of perioperative chemotherapy as a standard approach for this patient population. Nevertheless, current neoadjuvant strategies face emerging challenges including low pCR rates and limited effectiveness in specific subgroups such as Borrmann type IV gastric carcinoma (5). Furthermore, while triple-agent regimens enhance preoperative treatment efficacy, their substantial toxicity profiles significantly compromise patient compliance during perioperative management, necessitating the exploration of new models in clinical practice. Given the limited efficacy of traditional chemotherapy for advanced GC, immunotherapy has emerged as a promising and innovative therapeutic strategy, drawing significant attention in the field.

The clinical trials including CheckMate 649 (14), KEYNOTE-859 (31), ATTRACTION-04 (15), ORIENT-16 (16), and RATIONALE-305 (32) have demonstrated clinically meaningful survival benefits when combining immune checkpoint inhibitors with conventional chemotherapy in advanced G/GEJ adenocarcinoma. There is a 28-44% reduction in mortality risk. These findings solidify immunotherapy as a standard in current multimodal management of advanced GC, particularly in PD-L1 positive populations. Given the benefit data for immune checkpoint inhibitors (ICIs) in the first-line treatment of advanced GC, it is worth exploring their efficacy in LAGC. The latest results from the MATTERHORN trial indicate that for locally advanced G/GEJ cancers, the incorporation of perioperative immunotherapy not only achieves a higher pCR rate but also demonstrates significant advantages in enhancing EFS and OS among patients with LAGC (33). In addition, the DANTE study demonstrated that the combination of perioperative immunotherapy and chemotherapy can significantly downstage the clinical presentation and achieve a higher pCR rate compared to chemotherapy alone in LAGC. This effect is particularly pronounced in patients with MSI-H or high CPS score (19). However, data from the KEYNOTE-585 (17) and ATTRACTION-05 trials (34) indicated that the incorporation of immunotherapy did not significantly impact overall survival in the general population. Nevertheless, subgroup analyses suggest that for patients with MSI-H or high CPS scores, the combination of perioperative immunotherapy and chemotherapy plays a crucial role compared to chemotherapy alone, as evidenced by both the pCR rate and survival outcomes. So it follows that the higher pCR rate and R0 resection rate did not translate to longer survival. To further improve the effectiveness of neoadjuvant therapy, combination with other treatment strategies, including other immune checkpoint inhibitors, and antiangiogenic agents, is necessary.

Tumor angiogenesis, acknowledged as a pivotal hallmark of malignant neoplasms including GC, plays an essential role in providing vital nutrients and oxygen to support tumor proliferation. VEGF and its corresponding receptor (VEGFR) system serve as principal molecular mediators driving this angiogenic process. The targeted therapies that specifically disrupt tumor-associated angiogenesis not only demonstrate potent tumor growth suppression capabilities, but have also emerged as groundbreaking treatment modalities in clinical oncology practice (35). The unique vascular structures in tumor tissues, such as distortion, leakage, incomplete basement membrane and incomplete coverage of surrounding cells, eventually lead to insufficient perfusion of tumor tissues, hypoxia, infiltration of immune cells and obstruction of drug delivery (36, 37). In particular, reports have shown that VEGF can inhibit the maturation of DCs and antigen presentation, ultimately leading to immunosuppression of tumor tissues and exerting a promoting effect on cancer (38). Proposed by Jain’s team in 2001, it is believed that an appropriate dose of anti-angiogenic drugs can temporarily “normalize” abnormal blood vessels, which is manifested as: increased pericytes coverage in tumor tissues, improved basement membrane integrity, and enhanced perfusion and oxygenation (39). The “normalization” of blood vessels in tumor tissues can further improve the infiltration and function of immune cells, promote the reorganization of the immune microenvironment (reduce the infiltration of Treg cells, promote the polarization of M1-type TAMs, and reduce the expression of PD-L1) (40–42). Studies have shown that anti-VEGFR2 combined with anti-PD-1 treatment can significantly enhance T cell function (43), which showed a synergistic effect with immunotherapy. The prospective phase III Dragon IV trial (NCT04208347) has revealed that a multimodal neoadjuvant protocol integrating immune checkpoint inhibitors (anti-PD-1), VEGFR2 inhibitors, and SOX chemotherapy achieved a 3.66-fold increase in pCR rates compared to chemotherapy alone (18.3% *vs*. 5.0%; p<0.001) in locally resectable G/GEJ adenocarcinoma (25). The trial results of Dragon IV indicated that the combination of immunotherapy with PD1 and chemotherapy was feasible and had a synergistic effect. At present, clinical application of VEGFR TKIs remain constrained by dual challenges of compromised therapeutic efficacy and dose-limiting toxicities. Although the Dragon IV trial has achieved promising results of chemotherapy combined with targeted and immunotherapy (the pCR rate was improved by 3.7-fold compared to chemotherapy alone), the combination of multiple drugs, especially VEGFR2 inhibitors and anti-PD-1 drug, increase the incidence of grade≥3 TRAEs (34% vs. 17%). Therefore, it is necessary to develop and optimize new strategies to enhance and improve the efficacy and toxicity of multi-drug mode therapy. Fruquintinib is an anti-angiogenic small-molecule agent independently developed in China. It acts as a potent and highly selective tyrosine kinase inhibitor, effectively suppressing tumor growth by binding to and inhibiting VEGFR-1, VEGFR-2, and VEGFR-3. Furthermore, fruquintinib offers several advantages, including minimal off-target toxicity, favorable resistance profiles, and notable therapeutic efficacy (44).A number of trials have confirmed that fuquinitinib monotherapy or combined immunotherapy has achieved effective results and tolerable adverse reactions in patients with MSS or MSI metastatic colon cancer (45–48). Currently, the results of FRUTIGA trial indicated that fruquintinib plus paclitaxel as a second-line treatment significantly improved PFS, in Chinese patients with advanced G/GEJ (49). A number of prospective first or second-line clinical trials of combination therapy with furoquininib have been in full progress (NCT05625737, NCT05024812, NCT06158919, NCT05177068). From these updated first-line treatment data, the ORR ranges from 60.9% to 68.6%, with a DOR exceeding 96.7%. Notably, the study by Ma et al. demonstrated that in patients with G/GEJ cancer who received conversion therapy with fruquintinib combined with sintilimab plus SOX, the R0 resection rate reached 100%, the pCR rate was 10.3%, and the proportion of patients achieving a tumor regression grade (TRG) 0–1 was 20.7% (NCT05177068). A multicenter, phase II, single-arm, open-label clinical trial (FRUTINEOGA) on the application of fuquinitinib combined with chemotherapy as neoadjuvant therapy has also been carried out and the results are expected (28).In addition to the superiority in efficacy, fuquinitinib combined with PD1 and chemotherapy also showed some advantages in safety evaluation compared with the Dragon IV study.

To our knowledge, this is the first clinical trial investigating the combination of fruquintinib, sintilimab, and the SOX regimen as perioperative therapy for locally advanced, resectable G/GEJ adenocarcinoma. The optimal treatment plan, treatment timing, efficacy evaluation and benefit population of neoadjuvant therapy for gastric cancer are still controversial. Our prospective, phase II clinical trial may provide more evidence for enriching neoadjuvant or perioperative treatment options for locally resectable gastric cancer. If this phase II clinical trial achieves the anticipated results, we will conduct a large-sample, multicenter, and prospective study to more comprehensively evaluate the efficacy, safety, and biomarker benefit population of the treatment regimen used in the phase II trial. This study has several limitations inherent to its design. As a single-arm phase II trial, the relatively small sample size and homogeneous population may constrain the external validity of the findings, underscoring the necessity for validation in larger, multicenter cohorts with broader demographic diversity. Due to the limited sample size (n=25), EBV, CPS and microsatellite subgroups may have insufficient statistical power. The absence of a randomized control group further precludes definitive conclusions regarding whether the observed clinical benefits are attributable specifically to the fruquintinib-SOX combination or reflect inherent selection biases. Additionally, the primary focus on short-term endpoints leaves the long-term oncological implications (including OS, recurrence patterns, and delayed treatment-related toxicities) unexplored, necessitating extended follow-up to fully characterize the regimen’s risk-benefit profile.
