# Supplementary material for: Fruquintinib and sintilimab plus SOX as perioperative therapy for locally resectable advanced gastric/gastroesophageal junction adenocarcinoma: study protocol for a prospective, single-arm, phase II clinical trial
Source: Front Immunol. 2025 Jul 29;16:1638316. doi: 10.3389/fimmu.2025.1638316 (PMC12339537; doi:10.3389/fimmu.2025.1638316)
Supplement: Supplementary file 1 [file Table1.docx]

**Supplement Methods**

**Biomarker analysis**

**In situ hybridization (ISH) of Epstein-Barr virus (EBV)-encoded RNA (EBER)**

The presence of Epstein-Barr virus (EBV) in tumor tissues was evaluated using in situ hybridization (ISH) targeting EBER-1 and EBER-2, which are widely recognized as the gold standard for detecting EBV in tumor cells. Deparaffinized tissue sections were subjected to EBER-ISH analysis. The EBER probe (EBER ISH Kit, Product No: ISH-7001) specifically hybridized with the target RNA sequences within cells or tissues, forming a stable hybrid complex. Subsequently, a horseradish peroxidase (HRP)-conjugated anti-digoxigenin antibody bound to the hybridized probe, forming an immunocomplex. HRP then catalyzed the conversion of the chromogenic substrate into a brown, insoluble precipitate known as DAB (3,3' diaminobenzidine). This reaction allowed for the microscopic visualization of EBV-specific RNA signals within the tissue sections. EBV-positive gastric adenocarcinoma samples were used as positive controls.

**PD-L1 expression assessment**

Baseline PD-L1 expression levels in tumor tissues were evaluated using PD-L1 immunohistochemistry (IHC) with the 22C3 pharmDx assay (Agilent Technologies, Carpinteria, CA, USA). PD-L1 expression was quantified using a Combined Positive Score (CPS), which was calculated as the total number of PD-L1–positive cells, including tumor cells, lymphocytes, and macrophages, divided by the total number of viable tumor cells, multiplied by 100. Tumor samples were considered PD-L1 positive if the CPS was ≥1.

**Mismatch repair (MMR) protein expression by IHC**

MMR protein expression was assessed via immunohistochemistry (IHC) using specific antibody clones (MLH1: ES05; MSH2: FE11; MSH6: EP49; and PMS2: EP51; Dako, Carpinteria, CA). A complete loss of expression (scored as 0+ in 100% of tumor cells) for any of the four proteins analyzed was interpreted as indicating deficient MMR function. Since MMR proteins function as heterodimers, tumors were further categorized and compared based on MSH2/MSH6 deficiency versus MLH1/PMS2 deficiency.
